# Reducing the WCET and analysis time of systems with simple lockable instruction caches

**DOI:** 10.1371/journal.pone.0229980

**Published:** 2020-03-19

**Authors:** Alba Pedro-Zapater, Juan Segarra, Rubén Gran Tejero, Víctor Viñals, Clemente Rodríguez

**Affiliations:** 1 Dpt. Informática e Ingeniería de Sistemas, Instituto de Investigación en Ingeniería de Aragón (I3A), Universidad de Zaragoza, Zaragoza, Spain; 2 Dpt. Arquitectura y Tecnología de Computadores, Universidad del País Vasco, País Vasco, Spain; 3 HiPEAC; Universidad Rey Juan Carlos, SPAIN

## Abstract

One of the key challenges in real-time systems is the analysis of the memory hierarchy. Many Worst-Case Execution Time (WCET) analysis methods supporting an instruction cache are based on iterative or convergence algorithms, which are rather slow. Our goal in this paper is to reduce the WCET analysis time on systems with a simple lockable instruction cache, focusing on the Lock-MS method. First, we propose an algorithm to obtain a structure-based representation of the Control Flow Graph (CFG). It organizes the whole WCET problem as nested subproblems, which takes advantage of common branch-and-bound algorithms of Integer Linear Programming (ILP) solvers. Second, we add support for multiple locking points per task, each one with specific cache contents, instead of a given locked content for the whole task execution. Locking points are set heuristically before outer loops. Such simple heuristics adds no complexity, and reduces the WCET by taking profit of the temporal reuse found in loops. Since loops can be processed as isolated regions, the optimal contents to lock into cache for each region can be obtained, and the WCET analysis time is further reduced. With these two improvements, our WCET analysis is around 10 times faster than other approaches. Also, our results show that the WCET is reduced, and the hit ratio achieved for the lockable instruction cache is similar to that of a real execution with an LRU instruction cache. Finally, we analyze the WCET sensitivity to compiler optimization, showing for each benchmark the right choices and pointing out that O0 is always the worst option.

## Introduction

Real-Time systems are increasingly present in the industry and the daily life. We can find examples in many sectors such as avionics, robotics, automotive, manufacturing, or air-traffic control. A real-time system consists of a number of tasks, which perform the required functionality. These tasks can be organized by priorities and they have to be scheduled in a way so that they satisfy their deadlines. In order to guarantee their correctness, Worst-Case Execution Time (WCET) and schedulability have to be analyzed. WCET depends on the hardware technical specifications and how it interacts with the running task, so a particular WCET is not valid for hardware other than the one analyzed. If schedulability fails, the system must be redesigned, repeating all previous procedures. Since this is complex, some approaches propose to apply it just to safety-critical tasks, and not to other tasks in the real-time system [1]. Thus, improving the time required for a part of this validation process might reduce very much the time required to design a real-time system. Also, a fast WCET analysis can be an alternative to parametric WCET analysis, which is usually quite limited on the parameters it supports [2].

Given a processor with fixed-latency components, the WCET of a single task could be calculated from the partial WCET of each basic block of the task. However, current processor organizations include variable-latency components in order to enhance performance on the average case. Variable-latency components are caches, pipelines, branch prediction, and other speculative mechanisms [3]. Although performance oriented platforms benefit from this, the analysis for hard real-time systems becomes more difficult.

One of the main challenges in the WCET analysis is the memory hierarchy [4]. Conventional cache behavior depends on past references and, for a precise analysis, it is necessary to know all the previous memory accesses in order to predict the latency of the current memory access. Depending on the replacement policy, such dependence usually implies either a rather high analysis time (e.g. LRU), or a high overestimation in the WCET when grouping the possible alternative execution events (e.g. domino effects in Pseudo-LRU) [5]. Focusing on LRU replacement, current WCET analysis methods are based on Abstract Interpretation (AbsInt) [6, 7], Implicit Path Enumeration (IPET) [8], or use them both [9]. Given the significant analysis time these methods require for the WCET analysis of systems with an instruction cache, it is not clear whether they can support the analysis of complex programs on systems including other hardware components such as data cache, prefetch, etc. For instance, although theoretically both AbsInt and IPET support data caches [8], as far as we know no study has thoughtfully evaluated them.

To reduce the analysis time, many studies propose using fully-lockable caches [10], present in processors of most manufacturers, such as Motorola (ColdFire, PowerPC, MPC7451, MPC7400), MIPS32, ARM (904, 946E-S), Integrated Device Technology (79R4650, 79RC64574), Intel 960, etc. These caches, on a miss event, request the missed line to the next memory level, but on arrival they send it to a line buffer, without keeping any copy. Therefore no replacement is needed, and all the storage and control devoted to implement replacement in conventional caches becomes unnecessary and is removed. Since the content is known and it does not change, the hit/miss computation is much easier and it does not depend on any previous memory access, so the WCET analysis is simplified. However, the challenge now is to determine the best set of instructions to lock in cache, additionally to perform its WCET analysis. Therefore, cache locking methods try to find contents so that they generate the minimum WCET when locked in cache. Depending on their flexibility regarding the loading and locking points, the distinct sets of contents to manage, and also how they address the analysis (heuristically, analytically, etc.), there are many approaches to this problem. *Static locking* methods perform a single selection of instructions to lock among *all the tasks* that run in the system, so that such set is fixed at system start-up [11]. On the other hand, *dynamic locking* methods perform one or more selections of contents per task. In general, dynamic locking performs better than static locking in terms of WCET [12]. Focusing on dynamic locking, let us use *single-content dynamic locking* to refer to those dynamic locking methods that select a single content per task, which is loaded and locked at every task context switch (e.g. [13]), and *multiple-content dynamic locking* to those methods that allow each task to load and lock cache contents at will during its execution (e.g. [14]). Two very interesting properties of single-content dynamic locking are, first, that its WCET analysis can be performed without losing precision by structure-based methods, whose solving is much faster, and second, that such methods also provide the optimal selection of contents to lock [13]. This allows to extend the WCET analysis so that it includes prefetch [15], data cache [16, 17], and even to analyze at the same time the *Worst-case Energy Consumption* (WCEC) in order to obtain a balanced WCET-aware–WCEC-aware trade-off [18]. On the other hand, multiple-content dynamic locking methods may improve the WCET, but they must decide the best code locations to perform the loading and locking of instructions, and the best selection of instructions to lock at each loading point. Usually, both these problems are addressed heuristically in order to limit their associated analysis time, so their results are not optimal and their required analysis time may be comparable to that required for the WCET analysis of an LRU cache [14, 19]. Other studies use genetic algorithms to address these problems [20].

Finally, some studies assume *partial set-level lockable caches*. For each cache set, these caches are able to track a variable number of non-locked lines sorted in LRU order, and lock the remaining ones [21, 22]. Control complexity and storage to support this fine-grained locking-replacement surpasses the abilities of conventional caches and, of course, that of fully-lockable caches. To the best of our knowledge, such hardware is not yet ready, and current cache designs are far from reaching this behavior. The high number of configurations supported by partial set-level lockable caches requires high analysis times. For instance, a convergence process consisting of two steps has been proposed [22]. The first step performs a WCET analysis assuming a conventional instruction cache which may have some locked lines. The second step test whether there is a new suitable line to lock, configuring the instruction cache accordingly for the next iteration of the convergence algorithm. However, none of these studies provides system-independent baselines (e.g. always-hit, always-miss), being difficult to interpret their results. Also, they do not compare with conventional caches. Furthermore, when comparing to completely locked caches they use a biased hardware, since they do not consider the line-buffer component, required for locked caches to work properly [11, 13–17, 19]. Nevertheless, in this paper we focus on simple cache structures and fast WCET analysis methods, so approaches towards partial set-level lockable caches are out of our scope.

As stated above, a short analysis time is so important that many times heuristic methods are preferred to analytical methods. However, analysis methods aimed at lockable caches have not thoroughly explored the potential of these systems for a fast analysis. Our goal in this paper is to reduce the WCET analysis time of tasks in presence of lockable instruction caches. This is achieved by developing DLock-MS, a multiple-content dynamic locking method. Essentially, it consists of two key improvements built on Lock-MS [13], a single-content dynamic locking method.

Our first contribution is an algorithm to translate the Control-Flow Graph (CFG) to a tree-based structure representing the WCET analysis problem, which allows us to use Lock-MS as a structure-based method. Such kind of methods are in general the fastest ones, since they do not use convergence algorithms nor overlapped flow problems. With our algorithm the WCET problem is organized as nested subproblems, which exploits common branch-and-bound algorithms of Integer Linear Programming (ILP) solvers, so that each subproblem is optimized for a fast resolution. In terms of efficiency, our proposed algorithm generates the tree-based structure in a single pass, and explores each branch a single time.

Our second improvement addresses the size limitation that single-content dynamic locking methods present, not only to surpass such limitation with better WCET results, but also focusing on a fast and efficient analysis. DLock-MS applies a loop-based heuristics for the placement of multiple loading and locking points for the instruction cache, and then lets the solver to find the optimal content to load and lock at each point. Additionally, regions where the locked contents are fixed can be processed as isolated subproblems, which accelerates even more the WCET analysis and solving time, and even enables computing in parallel (not addressed in this paper).

The rest of this paper is organized as follows. Section Structure-based WCET analysis describes our methodological context. Our two proposals are described in Sections CFG to tree transformation and Placement of loading and locking points. In Section Results we evaluate them. Finally, our Conclusions are presented.

## Structure-based WCET analysis

Initial ways of approaching the WCET analysis problem through static analysis considered alternatives such as path-based, IPET, and structure-based calculation [3]. The main limitation of path-based calculation is that it must explicitly represent the exponential number of paths that a task may contain. Structure-based calculation, as used in initial versions of Heptane [23], for example assumed a tree-structure representation of the CFG to perform a bottom-up calculation of the specific costs of each branch. Its main limitation arose when used on systems with conventional caches, since it cannot support their inherent context-based behavior. Nevertheless, such approach was probably the fastest one [3]. So, for certain targets, improved structure-based methods may be the most adequate ones. Specifically, our contributions are applied on the WCET analysis method Lock-MS, briefly described below [13].

Lock-MS is a path-based/structure-based static WCET analysis method. Contrary to former structure-based methods, it does not calculate the WCET by a bottom-up traversal of the tree representation of the CFG, but generates an ILP model to be solved. Although such ILP model may be path-based or structure-based, in this paper we address the structure-based model. So, its application may be closer to IPET, which also generates an ILP model. However, IPET represents the WCET analysis as an ILP flow problem, whereas Lock-MS represents it as a structure-based ILP problem. In this paper only the main structure-based constraints are shown in examples, but modeling details of these methods can be found in previous work [8, 13].

Lock-MS is designed for the WCET analysis of systems with a fully-lockable instruction cache. So, additionally to obtain the WCET, it obtains the single content to load and lock into cache. Note that such approach is not compatible with IPET, since IPET maximizes possible execution cases and Lock-MS obtains the locked contents that minimize the WCET. That is, it performs a WCET-aware optimization regarding the contents to lock into the instruction cache. In order to model such behavior, Lock-MS associates each instruction memory line to a binary variable representing its presence in the locked cache. The cost of executing the task to analyze is then expressed as a set of linear constraints dependent of previous binary variables. Thus, solving the resulting ILP model provides both the optimal selection of instructions to lock into the instruction cache, and the resulting WCET.

An important detail to notice is that all solutions that comply with the IPET model are unsafe, except the one that maximizes the WCET. This is due to the space of valid solutions (] − ∞, *WCET*]) in the maximization problem. On the contrary, any solution complying with the Lock-MS model is safe, since valid solutions are the complementary ones in a minimization problem ([*WCET*, ∞[). For instance, a bad but safe solution would be to use an empty locked cache, which would provide an overestimated WCET. This implies that using Lock-MS, one may choose to stop the ILP solver if it takes too long, and safe results (suboptimal cache configurations and their associated WCET) are obtained even when the analysis is not completed.

Finally, it must be taken into account that any contextual information such as path taken, relation between variables, unfeasible paths, hardware state (cache, buffers, branch predictors, etc.) is problematic in WCET analysis, and it usually implies overestimating the WCET. This is also the main limitation of structure-based methods. Such contextual information depends on the hardware to analyze. For instance, this limitation discourages from using Lock-MS on conventional caches, since the contextual information for such systems is the whole cache state. Nevertheless, Lock-MS can incorporate the contextual information required for other hardware such as a locked cache plus line-buffer (single-line conventional instruction cache) [13], an instruction prefetch buffer implementing a *next-line tagged* prefetch policy [15], and the predictable data cache ACDC [16, 17]. The context for an accurate analysis of such hardware is smaller, and it can be integrated into the structure-based model along with the CFG. However, no algorithm to transform the CFG into a tree structure (required for any structure-based method) has been proposed [13, 23].

## CFG to tree transformation

Our first contribution is an algorithm for efficiently translating the CFG information into a tree structure. First, loops and functions other than the main program are substituted by virtual nodes (basic blocks) in the CFG. Then, they are processed as independent sub-CFGs to be transformed into independent trees. For the main CFG and each one of the sub-CFGs, Algorithm 1 is applied recursively from the starting node in the corresponding CFG and an empty starting path (*Explore*(*start*,∅)). This algorithm performs a recursive in-depth search that builds the trees associated to each CFG and generates their corresponding ILP constraints, according to the Lock-MS model. Essentially, each tree is composed of a conditional node plus all its alternative paths until reaching another conditional node. Algorithm 1 works as follows. Lines 1 and 2 address the ending nodes of a CFG, returning just the ongoing path plus the current (ending) node and completing the exploration of such path. In lines 3 and 4, corresponding to a node with a single child, the exploration just goes deeper by following this single child in the path. Lines 5 and 6 correspond to the exploration of a conditional node (more than one child) already explored. Since it is already explored, its corresponding processing and constraint generation have already been performed, so further exploration of this node is not required. Finally, lines 7 to 13 describe how to proceed when the current node is a conditional node that has not yet been explored. In such case, a new tree is generated, with the current node as its root. Each child of the current node is explored to form the branches (alternative paths) of this root (lines 8 and 9). Then, each branch is processed, and the corresponding ILP constraints (explained below) are set (line 10). Finally, the current node is set as explored, returning the path until this node (lines 12 and 13). It must be taken into account that the entry node of a loop is not considered as a child of the nodes with the back edges.

**Algorithm 1** Explore(*currCFGnode*, *currPath*)

1: **if** |children(*currCFGnode*)| = 0 **then**        # no more nodes in path

2:  **return** *currPath* + *currCFGnode*

3: **else if** |children(*currCFGnode*)| = 1 **then**     # single child: expand path

4:  **return** Explore(child(*currCFGnode*), *currPath* + *currCFGnode*)

5: **else if** |children(*currCFGnode*)| > 1 **and** explored[*currCFGnode*] **then**

6:  **return** *currPath* + *currCFGnode*      # already explored conditional

7: **else**         #unexplored conditional (|children(*currCFGnode*)| > 1)

8:  **for all** *childNode* ∈ children(*currCFGnode*) **do**    #process each alternative path

9:   *alternativePath* ← Explore(*childNode*, *currCFGnode*)

10:   processAndBuildConstraint(*currCFGnode*, *alternativePath*    #set constraints like *C*_*BBx*_ ≥ *alternativePath*

11:  **end for**

12:  explored[*currCFGnode*] ← *true*

13:  **return** *currPath* + *currCFGnode*      #all alternative paths processed

14: **end if**

**Fig 1:**
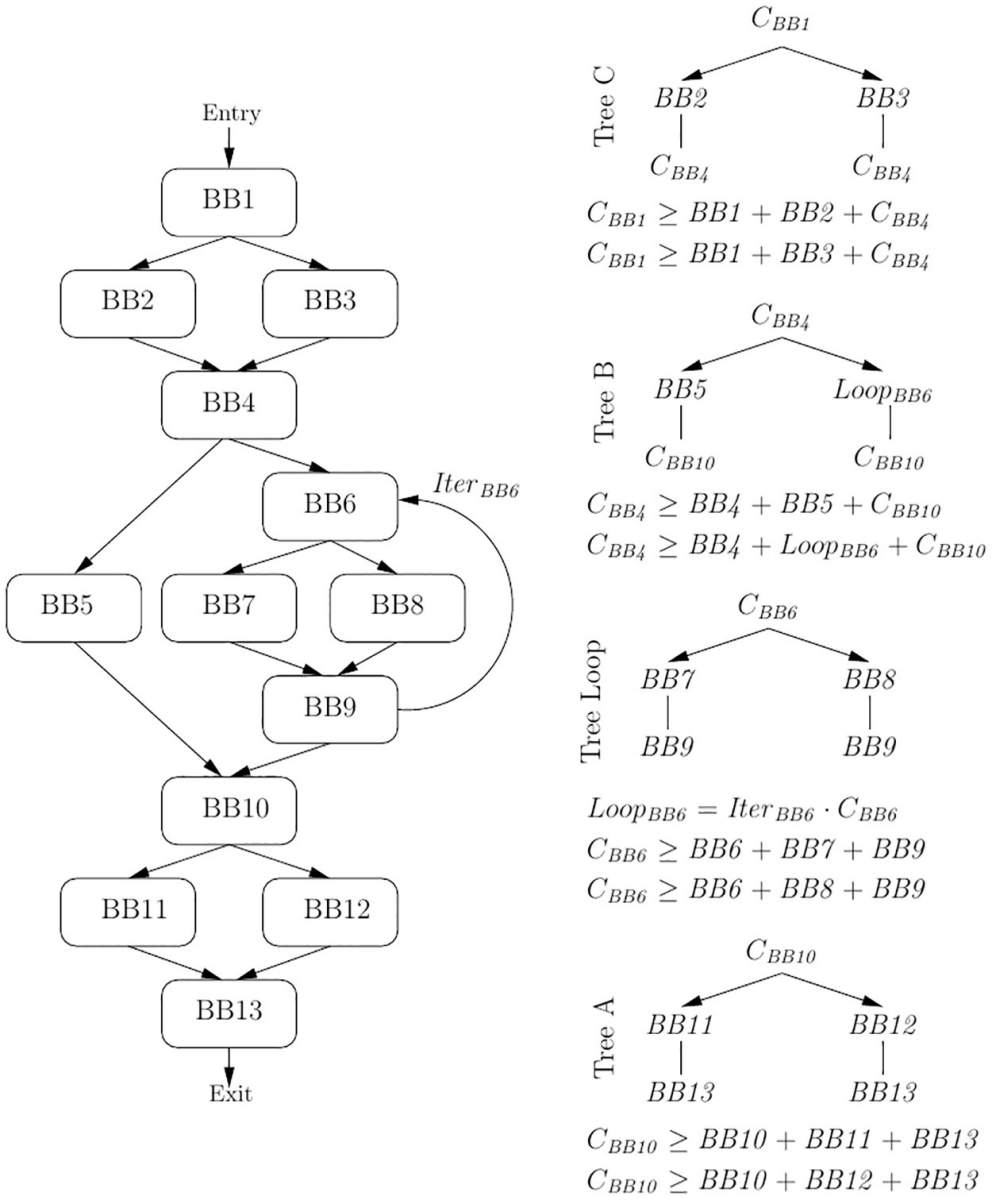
Simple CFG example, its associated trees, and their ILP constraints.

Essentially, the ILP constraints (line 10, Algorithm 1) model a minimization problem for a tree of nodes (basic blocks, BB). The cost of each particular node BB*i* would correspond to the total cumulative cost of executing the node, which would be an expression depending on its different execution cases and their number of occurrences. For each cacheable memory line *j* in the basic block, an associated variable *cachedBBi*_*j*_ determines whether it should be cached or not in order to reduce the WCET. Such cached memory lines cannot grow beyond the cache capacity, so they are also constrained according to the cache sets and ways. For instance, assuming just a lockable cache and a basic block BB1 fiting in a single cache line, these costs would be set as *BB1* = *hitCostBB1* ⋅ *nExecsBB1* ⋅ *cachedBB1* + *missCostBB1* ⋅ *nExecsBB1* ⋅ (1 − *cachedBB1*), where *hitCostBB1* and *missCostBB1* would be precalculated constants based on the hardware parameters, *nExecsBB1* would depend on the CFG, and *cachedBB1* would be a logical (0/1) variable. Nevertheless, in this paper we focus on the structure of the general constraints modeling the CFG, and not in those modeling the hardware [13, 15–18]. As an example, Fig 1 shows a CFG with 12 explicit paths to consider for the WCET analysis, the resulting trees after its transformation, and their corresponding main ILP constraints. Notice that with a locked cache the worst case of executing a loop cannot include combinations of the alternative paths that it contains [13]. So, the 12 paths to consider execute the following basic blocks: 1-2-4-5-10-11-13, 1-2-4-5-10-12-13, 1-3-4-5-10-11-13, 1-3-4-5-10-12-13, 1-2-4-6-7-9-10-11-13, 1-2-4-6-7-9-10-12-13, 1-2-4-6-8-9-10-11-13, 1-2-4-6-8-9-10-12-13,1-3-4-6-7-9-10-11-13, 1-3-4-6-7-9-10-12-13, 1-3-4-6-8-9-10-11-13, and 1-3-4-6-8-9-10-12-13. Tree A represents the latest conditional, in BB10, and its cost would be the maximum of its two alternative branches. Tree B represents from BB4 until BB10, which has already been explored. The loop starting in BB6 is analyzed as an independent CFG with its own tree (Tree Loop), and it is represented as a virtual node (*Loop*_*BB6*_) in Tree B. Tree C represents BB1 to BB4. Thus, instead of the 12 paths in the CFG, our proposal provides 4 subtrees with 2 subpaths each one. The WCET can be set as the cost of the whole tree, i.e. the cost of its root: *WCET* = *C*_*BB1*_. In turn, the cost of each tree (composed of costs of nodes and subtrees) must be greater than any of its alternative paths, as detailed in Fig 1. Then, *minimizing* the variable *WCET*, the ILP solver would provide the best values for the *cachedBBi*_*j*_ variables, i.e., those that provide the minimum WCET.

Apart of the actual CFG to tree transformation, our approach has the following benefits. All the process is performed in a single pass, and no preprocessing is required to known the size of the CFG. Also, branches are explored just once. Since branches are usually associated to basic blocks, this makes our approach essentially linear with respect of the basic blocks in the program. Only terminating basic blocks may be explored more than once (e.g. loop-terminating BB9 and CFG-terminating BB13 in Fig 1).

## Placement of loading and locking points

The main drawback of single-content dynamic locking methods such as Lock-MS is their inability to follow the working set changes that could appear during program execution. So, an apparently straightforward improvement is to define region-specific contents to load and lock previously to the execution of such regions in the task [14, 19, 24]. However, this implies finding both adequate loading points and adequate instruction lines to lock at each point, accounting for the time overhead of instruction loading, and modifying the memory layout of the task. Thus, improving the behavior of the optimal single-content dynamic locking is not as straightforward as one could think. Indeed, studies claiming better results than single-content dynamic locking specifically state that they remove the line-buffer hardware component, required for locked caches to work properly [21].

Our second contribution is a dynamic locking heuristics that addresses the problems stated above. We consider the entry of outer loops as the points to load and lock the instruction cache. This simple heuristics addresses the following points. First, each program region benefits from a privately locked cache content. This means that our analysis finds the contents that minimize the WCET for each region. Second, there are no loading and locking points inside loops, so that their corresponding overhead will never be multiplied by loop iterations. Third, since locked caches exploit temporal reuse, and temporal reuse is found in loops, our heuristics allows to use the whole cache capacity for each of these temporal reuse environments. Note also that such heuristics does not prevent to set other loading points. That is, the resulting ILP model can be modified if the designer wants to change their location.

**Fig 2:**
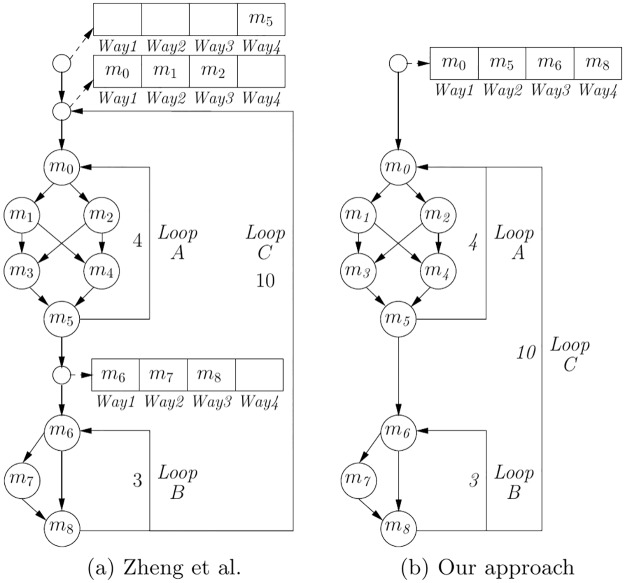
Locked basic blocks and loading points on the example presented in [22]. A: the proposal of Zheng et al. B: our approach.

In order to have a practical insight, let us compare our proposal with other approaches. Fig 2A reproduces the example presented in a recent study to show the improvement of its approach with respect to previous work (Fig 3 in [22]). As it can be seen, it sets three loading and locking points with specific contents (dashed arrows). Instead, our proposal (Fig 2B) sets a single loading and locking point with the optimal contents for the outer loop. In order to perform a simple WCET calculation, let us just account for hits and misses considering that each loaded cache line is equivalent to a miss (*M*_*i*_). For the loading and locking points, a function call (with its corresponding executed instructions and cache misses) is assumed [22]. So, for our proposal to be in disadvantage, let us consider that the cost of each loading point is just that of a single miss (*M*_*p*_). In such case, the obtained WCET for Fig 2A would be: (*M*_*p*_ + *M*_5_) + 10 · (*M*_*p*_ + *M*_0_ + *M*_1_ + *M*_2_) + 4 · (3*H* + 1*M*) + (*M*_*p*_ + *M*_6_ + *M*_7_ + *M*_8_) + 3 · (3*H*)) = 122*M* + 210*H*. On the other hand, with our proposal (Fig 2B) the obtained WCET would be: (*M*_*p*_ + *M*_0_ + *M*_5_ + *M*_6_ + *M*_8_) + 10 · (4 · (2*H* + 2*M*) + 3 · (2*H* + 1*M*)) = 115*M* + 140*H*. So, our approach would provide a significantly lower WCET (5.7% fewer misses and 33.3% fewer hits). Nevertheless, it is important to remember that the heuristics of the proposal in Fig 2A requires a partial set-level locking cache, whereas ours assumes just a fully-lockable cache. Since the hardware is completely different, this means that our methods are not comparable, except when the whole cache is locked, as in Fig 2. Also, note that other approaches must perform preliminary or convergent WCET analyses in order to set their loading points, which imply much longer analysis times [19, 22].

**Fig 3:**
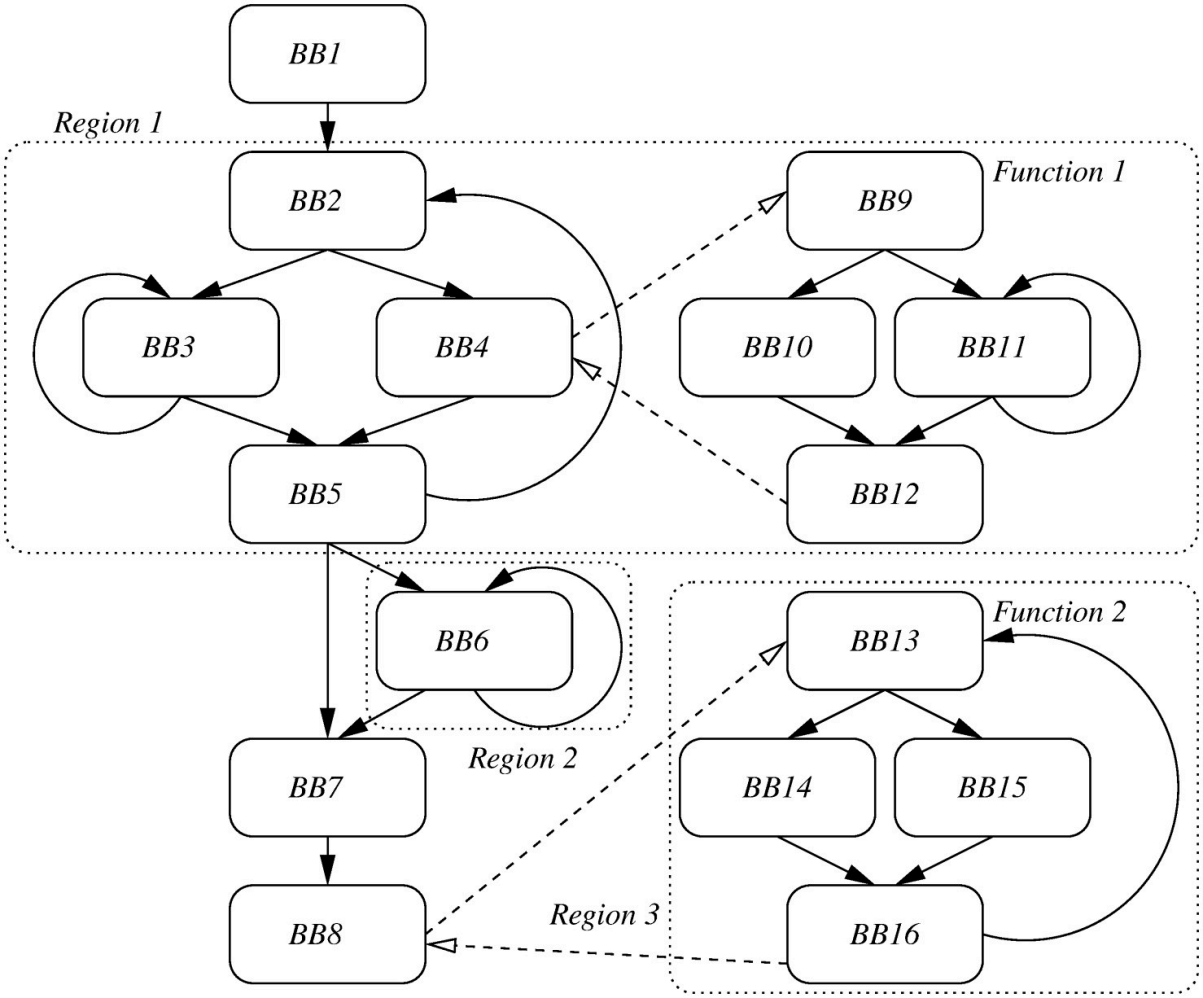
CFG example with the regions considered in DLock-MS.

As stated above, each outer loop is associated to a region (with specific and fixed cache contents) which is analyzed isolatedly. Fig 3 shows an example with five loops, the outer ones set as regions. The instructions in basic blocks (nodes) that do not belong to any region (instructions in BB1, BB7, and BB8) are not candidates to be locked, but they will be cached nevertheless by the line-buffer [11]. In this way, each region can be addressed as a single-content dynamic locking isolated problem. Such approach ensures that, for each loading point, the selected cache contents are optimal. That is, the ILP solver provides the selection of the contents for each loading point so that the execution cost for the corresponding region in the worst case is the minimal possible one. Also, the number of paths to explore is reduced even more. Discarding loops, the number of paths to explore in Fig 3 without applying regions would be 3 × 2 × 2. Since analysis of regions is isolated, with our dynamic locking heuristics the number of paths to explore is 3 + 2 + 2. Note also that having independent regions allows us to build an ILP problem for each region, solve all of them in parallel, and then use their result (the partial WCET of each region) as the specific cost for this region in the main ILP model. As far as we know, no other method exhibits such potential. In this paper we do not exploit such parallelism, but it would reduce even more the analysis time.

## Results

In this section we evaluate DLock-MS, our extension of Lock-MS, both in terms of the required analysis time and the effectiveness of the results. It is studied for the different optimization levels in compilation, which may transform loops in different ways. Indeed, several interesting observations have been found on such experiments, so a specific evaluation of the impact on the WCET of the optimization levels in compilation and their loop transformations is also included in this section.

Our target architecture is a 32-bit ARM processor considering the default architecture assumed by the Otawa toolset [9], a state of the art tool based on AbsInt and IPET. That is, a single instruction wide pipeline with stages consuming no more than a single cycle (apart of instruction cache misses) and a static branch prediction of *not-taken*. This means that all taken branches introduce a penalty of 2 cycles. Also, we assume the presence of an instruction line buffer, i.e. a single-line conventional (dynamic) instruction cache, as in previous studies [11, 15, 16]. Such line buffer is required for a lockable instruction cache to work properly. The tested binaries have been generated with ARM Compilation Toolchain *GCC 6.3.1* [25] with hardware support for floating point. For each benchmark, experiments include all possible combinations of the parameters shown in Table 1. We have carried out further experiments varying the compiler version, line size, memory latency, and cache associativity, but they did not show any interesting or unexpected behavior, so they are not included in this paper. We consider an instruction cache hit cost of 1 cycle, and a relatively low memory latency (10 cycles). Other studies consider off-chip main memories and assume a higher miss penalty, such as 30 cycles [22]. Clearly, improvement in such systems would be much higher.

**Table 1:** Experimental setup.

Parameter	Tested cases
*Analysis method*	Lock-MS, DLock-MS, Otawa (AbsInt+IPET), simulation
*Max. iterations in loops*	Manually set
*Compilation toolchain*	ARM gcc 6.3.1
*Optimization level (-O)*	0, 1, 2, 3
*Instr. line buffer*	Present (cache line size)
*Instr. cache line size*	32 Bytes
*Instr. cache assoc*.	Direct-mapped, 2 set-associative
*Instr. cache size*	128, 256, 512, 1024 Bytes, always-hit, always-miss
*Memory latency*	10 cycles
*Data memory access*	Ideal (no penalty cycles)

We have implemented DLock-MS as a module on the Otawa framework [9]. Such framework provides us with the parsing of binaries and the CFG, so that we can directly apply our proposed CFG to tree transformation, and build our required ILP constraints from the tree structure. Also, Otawa requires control flow information, such as loop bounds, to be provided in advance. For each binary, this information has been manually set by carefully studying the effect of compiler optimizations, particularly loop transformations such as unroll, fusion, split, move, duplicate, etc. Nevertheless, any loop bound analysis tool could be used [26, 27]. The only transformation we have found is full loop unrolling at optimization levels O2 and O3 in benchmarks *binarysearch*, *crc*, *g723_enc*, *lift*, *matrix1*, *md5*, *ndes*, and *petrinet*.

Table 2 shows the 30 benchmarks used in our experiments, downloaded in feb. 2017 from the TACLeBench [28] and Mälardalen [29] suites. Some benchmarks in these suites have been discarded for the following reasons: compilation errors (*powerwindow, bitcount, gsm_dec, rijndael_dec, dijndael_enc, susan*), unknown number of loop iterations in library functions (*powerwindow, prime, adpcm_dec, adpcm_enc, ammunition, anagram, cjpeg_transupp, cjpeg_wrbmp, epic, huff_enc, rijndael_dec, rijndael_enc*), and CFG extraction issues. CFG extraction issues include switch constructs as jumps to unknown addresses, CFGs with irreducible loops (i.e. loops with multiple entries), recursive functions, etc. These issues come from binary parsing limitations, but they do not affect our proposal. Benchmarks with such issues are *sha, gsm_enc, h264_dec, cover, duff, mpeg2, lms, test3, quicksort, recursion*. For different benchmarks containing the same algorithms (*binarysearch/bs*, *countnegative/cnt*, *jfdctint/fdct*, *petrinet/nsichneu*), only the TACLeBench version is shown in our results, although both versions have been tested to confirm that each pair shows the same behavior.

**Table 2:** Tested benchmarks (TACLeBench [28] and Mälardalen [29]).

Name	Binary code size (Bytes)				O3 complexity (Paths)		
	O0	O1	O2	O3	CFG	Tree	T+R
*audiobeam*	8572	3880	3940	4848	∼ 2^597^	2^24^	1512
*basicmath*	10048	4516	4516	5028	> 2^2080^	2^62^	69
*binarysearch*	604	264	260	1812	16	2	2
*bsort*	652	276	200	200	2	2	2
*complex_updates*	936	428	376	2300	1	1	1
*countnegative*	780	356	300	320	1	1	1
*crc*	1120	448	384	580	256	256	256
*dijkstra*	1544	764	636	808	> 2^1860000^	124	63
*fft*	1644	960	836	784	> 2^2048^	4	3
*filterbank*	1652	768	688	880	2	2	2
*fir2dim*	1456	712	640	1148	1	1	1
*fmref*	6344	3156	3288	5892	> 2^854^	2^52^	234995
*g723_enc*	6772	2724	2960	6104	-	-	-
*iir*	748	348	340	556	1	1	1
*janne_complex*	264	100	100	100	1	1	1
*jfdctint*	2972	1088	1136	1136	1	1	1
*lift*	3828	2440	2304	3704	> 2^1001^	4462	4462
*ludcmp*	2540	1084	972	4004	∼ 2^95^	2789600	776
*matmult*	704	304	324	308	1	1	1
*matrix1*	592	264	244	380	1	1	1
*md5*	7968	3268	3128	5432	> 2^2827^	2^31^	78839
*minver*	3048	1252	1108	1920	∼ 2^30^	1800	363
*ndes*	3168	1464	1760	2296	∼ 2^998^	98560	104
*petrinet*	6644	3700	3604	3644	> 2^133^	2^67^	2^67^
*pm*	8092	3648	3692	3984	> 2^2541^	2^33^	374641
*qsort-exam*	1480	620	620	620	∼ 2^175^	40	40
*qurt*	1092	580	548	540	1000	1000	1000
*select*	1332	516	520	520	∼ 2^64^	30	16
*st*	1680	768	780	1044	162	162	162
*statemate*	9508	7600	6676	6388	> 2^1000^	2^46^	2^46^

Values under *Binary code size (Bytes)* in Table 2 show the number of bytes of the analyzed instructions in the binary file, depending on the optimization level. That is, the sum of all instructions in the CFG times their size (4 bytes per instruction). Optimization for size (-Os) has been discarded due to it generally requires library functions containing loops with an unknown number of iterations. For the O3 optimization, we also show several values to provide an insight of the complexity of the WCET analysis regarding the number of execution paths to analyze. Column *CFG* shows an approximation of the number of paths in the CFG. Column *Tree* shows the specific number of paths to analyze with a structure-based WCET analysis method. Such value corresponds to the application of our first improvement. Finally, column *T+R* shows the paths corresponding to the application of our regions on the tree, i.e. our DLock-MS method. In this case, the reduction in the number of paths is due to each region is analyzed in isolation. This means that the number of paths of consecutive regions are not multiplied, but added, as explained on Fig 3. Note also that counting the number of paths may be harder than performing the WCET analysis, since most analysis methods merge paths precisely to avoid their combinatorial explosion. For instance, we have been unable to obtain the number of paths in the O3 version of the *g723_enc* benchmark.

When applying DLock-MS, some overhead cost must be considered. We assume that dynamic locking is performed by executing functions, as other studies do [22]. These functions load and lock into the cache the set of instructions required for the next region of code. Specifically, we consider a penalty of 47 cycles per call, one per region, which would correspond to the execution costs, cache misses, and pipeline penalties of an estimated length of code of 12 instructions. Additionally, for each locked cache line, we add the cost of the memory latency (10 cycles). This is a conservative scenario, since lock instructions might use specialized load operations, e.g. loading a large number of contiguous memory lines using some kind of burst memory transfer mode, which could decrease notably the total transfer time [22].

Our resulting ILP models are solved by *lp-solve* version 5.5.2.3. Due to the particular nesting properties of our structure-based model, we have used the solver options -BB, -Bc, -Bd, -Bg, and -Bo to order variables and apply a greedy reverse branch-and-bound.

### Evaluation of analysis times

In this section we study the time required for the WCET static analysis of DLock-MS, an extension of Lock-MS with an efficient CFG to tree transformation and a dynamic-locking heuristics.

**Fig 4:**
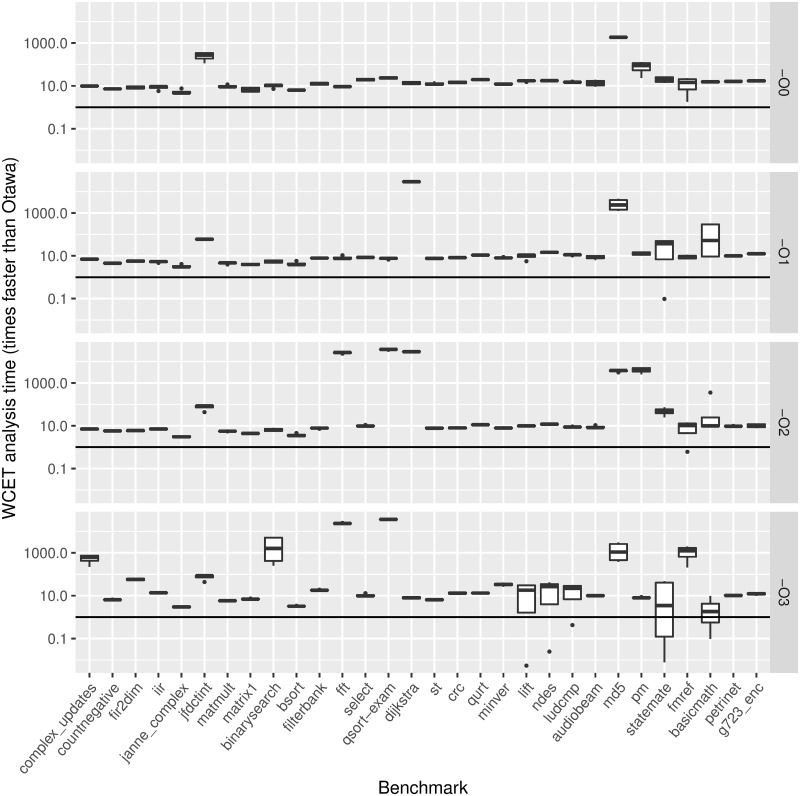
Comparison of WCET static analysis time of our approach (structure-based) and Otawa (Abstract Interpretation + IPET).

Fig 4 shows our analysis times compared to those required by the WCET analyzer (AbsInt + IPET) in Otawa (owcet v1.2.0) [9]. The *x* axis shows the benchmarks, and the *y* axis represents the attained WCET analysis speedup: execution time of the Otawa WCET analysis assuming a conventional LRU instruction cache divided by execution time of DLock-MS on a lockable instruction cache of the same size. This is shown for each optimization level, specified on the right side. Benchmarks are ordered by the number of paths to explore in the tree-based representation of the O3 optimization, to provide some insight of their complexity. For each benchmark, we show with boxplots all experiments performed varying the cache size (see Table 1). That is, each column shows a box whose limits indicate the first and third quartiles, with a mark inside showing the median. Vertical lines outside the box show the variability outside these quartiles, and points beyond these lines indicate outlier values, i.e. results that are statistically not relevant. Also, the horizontal line shows the unit speedup. Thus, the higher the boxplots, the faster is our WCET analysis compared to that of Otawa.

Regarding the analysis time, we have restricted it to 10 minutes, so that any experiment taking more than such time is assumed to take exactly 10 minutes. When such cases appear in Otawa, note that we are assuming an analysis time lower than it should be. With DLock-MS, only one experiment takes more than 10 minutes. However, it is important to note that we are able to provide safe WCET results before our WCET analysis/optimization is completed. That is, when we stop our analysis at 10 minutes, we already have a safe WCET bound and the specific sets of contents to load at each locking point, although we cannot guarantee that it is the optimal configuration, i.e. the one that provides the lowest WCET. So, assuming that experiments cannot take more than 10 minutes benefits Otawa in the comparison.

From Fig 4 we see that boxes are just horizontal lines in many cases. For such benchmarks, this means a very low speedup variability against cache size. Across all benchmarks and optimization levels, DLock-MS is commonly around 10 times faster than Otawa, although certain benchmarks may be especially difficult to analyze for each method. For instance, results above 10^4^ correspond to analyses not completed by Otawa in 10 minutes. On the other hand, the few slowdowns that appear are due to the existence of several paths with very similar or equal execution times, preventing the solver from discarding them early, as in the *statemate O3* case. Also, it must be taken into account that Otawa is faster than other approaches. For instance, the required analysis time reported in other studies is more than 30 times longer than ours [22].

Although not shown in Fig 4, we have also studied the time DLock-MS spends on each part of the analysis. In the experiments shown in this figure, our approach takes an average of 0.06 seconds to obtain the CFG and generate the tree structure and the ILP constraints, whereas the ILP solver takes an average of 2.29 seconds to solve the problem.

### Evaluation of effectiveness

**Fig 5:**
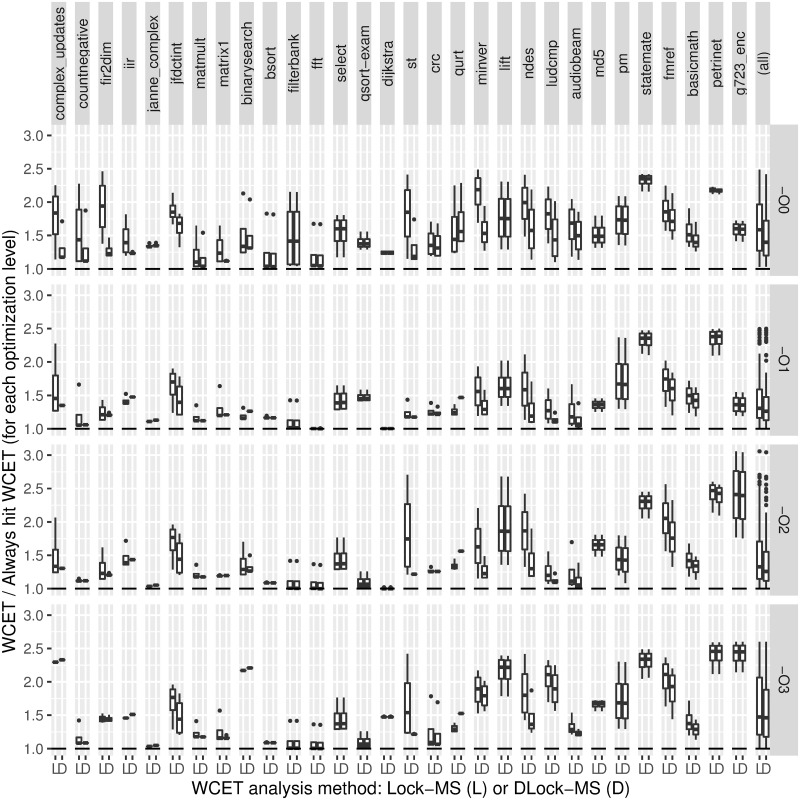
Comparison of the WCETs of Lock-MS and DLock-MS, the lower the better. Lock-MS is the single-content dynamic locking method baseline, and DLock-MS incudes our proposals to provide a multiple-content dynamic method. The data points forming the boxes correspond to the configurations defined by the cartesian product of the parameter values in Table 1.

Fig 5 shows the evaluation of the effectiveness of DLock-MS compared to the former Lock-MS method. As above, results are presented by boxplots for each optimization level. Since DLock-MS extends Lock-MS essentially by supporting multiple loading and locking points, in general its results should be equal or better. Specifically, WCETs are 2.2% better in average, including the extra cost introduced by the locking points. Such overhead (a function call for each locking point) supposes 2.1% of the WCET in average. Applying a one-sided Fisher Sign Test with a confidence level of 0.99), a *p*-value of 2.2 ⋅ 10^−16^ is obtained, stating that DLock-MS performs better than Lock-MS in terms of objective values.

Although improvements in Fig 5 may seem small, note that in many cases Lock-MS may reach the best possible WCET (e.g. *dijkstra*), so improvements may be impossible. Also, adding unnecessary loading and locking points may slightly increase the WCET, although this increment should be only noticeable for simple benchmarks (e.g. *iir*, *janne_complex*, *binarysearch*). For more complex benchmarks (those on the right side), WCET improvements are clearer, and the only problematic case is that of several regions loading the same contents, as in *qurt*. Nevertheless, note that such situation is trivial to detect and avoid.

**Fig 6:**
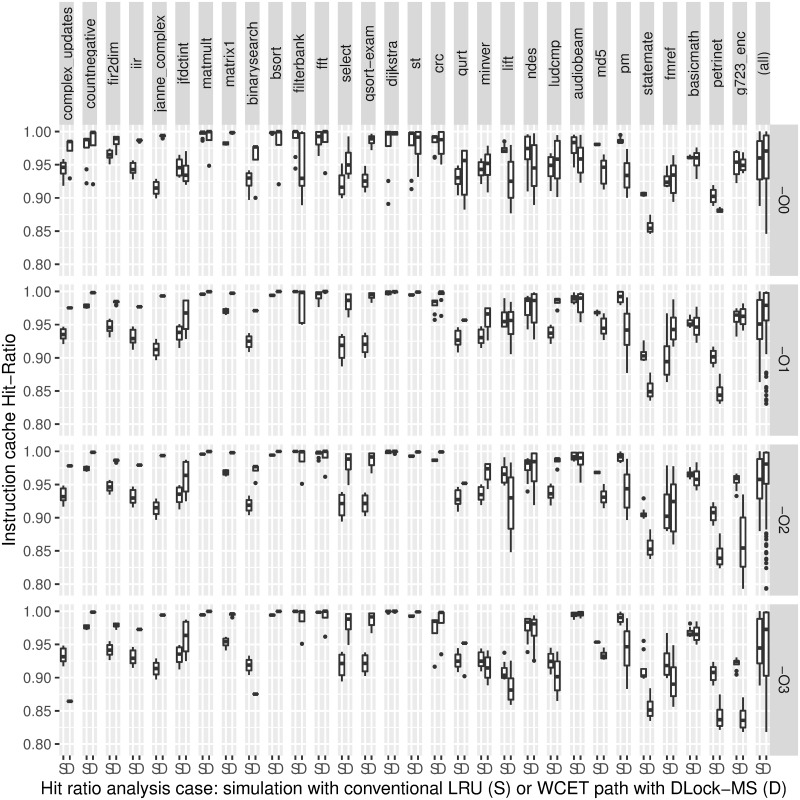
Comparison of the instruction cache hit ratios of our approach (hit ratio achieved throughout our analyzed WCET path) and an execution simulation (hit ratio of an execution simulation with a conventional LRU instruction cache), the higher the better.

Additionally to the previous comparison, let us also evaluate the effectiveness of the lockable instruction cache when analyzed with DLock-MS. Since the WCET decreases linearly with the instruction cache hit ratio, if our hit ratio in the WCET path is similar to that of a real execution, we can ensure that our results are accurate enough. Moreover, in this way we avoid that peculiarities of WCET analysis methods blur the actual goal, i.e. being slightly better than the WCET bound of other methods is far less important than being close to the actual WCET of a program. Furthermore, we compare our lockable instruction cache results to an LRU instruction cache, in order to test that DLock-MS reaches an acceptable performance. For obtaining actual hit ratios we use the Gem5 v2.0 simulator [30] configuring an equivalent pipeline with an LRU instruction cache of the same size. On the other hand we have obtained the hit ratio of the locked instruction cache in the worst-case execution path when applying DLock-MS. As above, results are presented by boxplots for each benchmark and optimization level. Fig 6 shows that hit ratios in the worst-path with a lockable cache are comparable to those with an LRU cache across all benchmarks and optimization levels, and the average is always better for the dynamic locked cache (rightmost column). In fact, there are many cases where the dynamic locked cache outperforms the LRU cache. This means that, for many benchmarks, locking the right code is likely to perform as good or better than an LRU policy, whose dynamism may evict content that will be used soon again. On the other hand, the benchmarks whose LRU hit ratio is higher are mostly located on the right side of Fig 6. This is consistent, since the natural dynamism of an LRU cache adapts its behavior to such larger and more complex benchmarks, whereas a lockable cache, even with a dynamic locking heuristics, has a more restricted behavior.

### Impact of compiler optimization level

Most studies on real-time systems disable optimizations for an easier high-level/binary code matching and, as far as we know, none of them has performed a thoroughly analysis regarding how optimizations affect the worst-case execution time. Intuitively, optimization reduces the average execution time, so in general it should also reduce the WCET. However, any optimization that reduces the average execution time by increasing the execution time of uncommon paths would increase the WCET if it is found through one of these uncommon paths. In this section we study the impact on the WCET of the optimization levels in compilation. We focus on the results for gcc 6.3.1, but results for gcc 4.8.4 (not shown) present almost identical trends.

**Fig 7:**
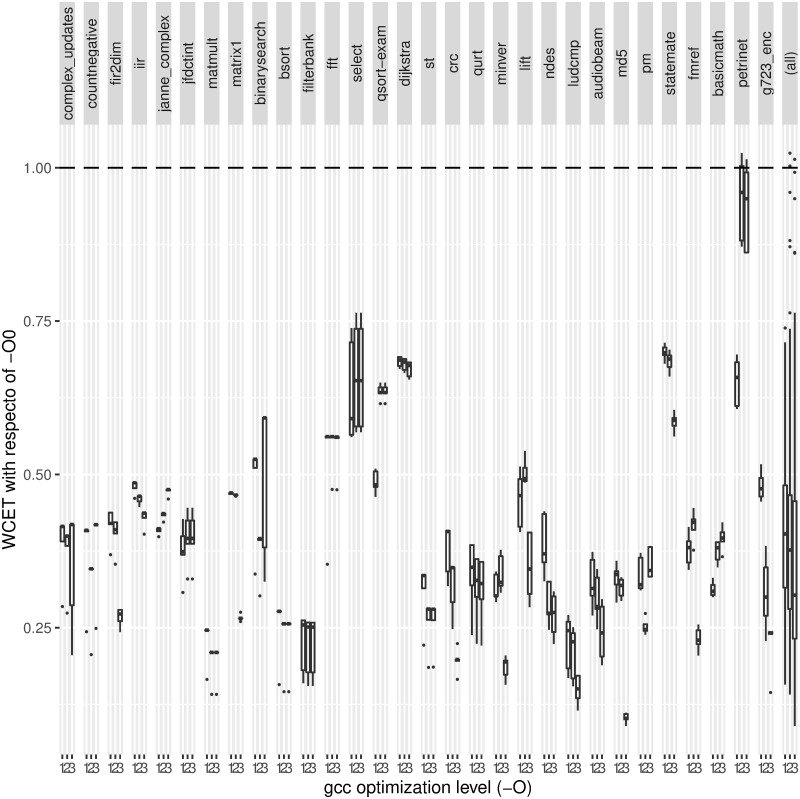
Effects of the compiler (gcc 6.3.1) optimization level on the WCET (the lower the better).

Fig 7 shows how the optimization level affects the WCET. The *x* axis shows the optimization levels 1, 2 and 3, and the *y* axis represents the WCET relative to compiling without optimizations (-O0). This is shown for each benchmark, plus the aggregated case on the right side, presented with boxplots as above. Also, an horizontal line is shown to mark the baseline (the WCET of the binary compiled without optimizations for each experiment) at *y* = 1. Thus, the lower the boxplots, the lower (better) the WCET for the corresponding optimization.

The most important observation is that, in average, boxplots are around 0.3. This means that, in general, the WCET of a given optimized code would be around one third of its WCET without optimizations. Hence, real-time systems should use optimized binaries. In average, the best results are reached by O3, although each benchmark may have a specific behavior.

Another interesting detail is that the improvement of WCET is not (exclusively) due to the size of the resulting binary code. O3 binaries are usually larger than those compiled with O1 and O2 (see Table 2), and nevertheless O3 binaries present a lower WCET. For instance the size of O3 binary codes is around twice the size of O2 in *fir2dim*, *fmref*, *g723_enc*, *iir*, four times in *ludcmp*, and six times in *complex_updates*, and nevertheless O3 results in a lower WCET. This is especially interesting, since all experiments conducted stress the cache. However, such optimizations may also turn into slightly adverse effects for the WCET, as can be seen in *binarysearch*, where O3 has around 7 times the size of O2.

**Fig 8:**
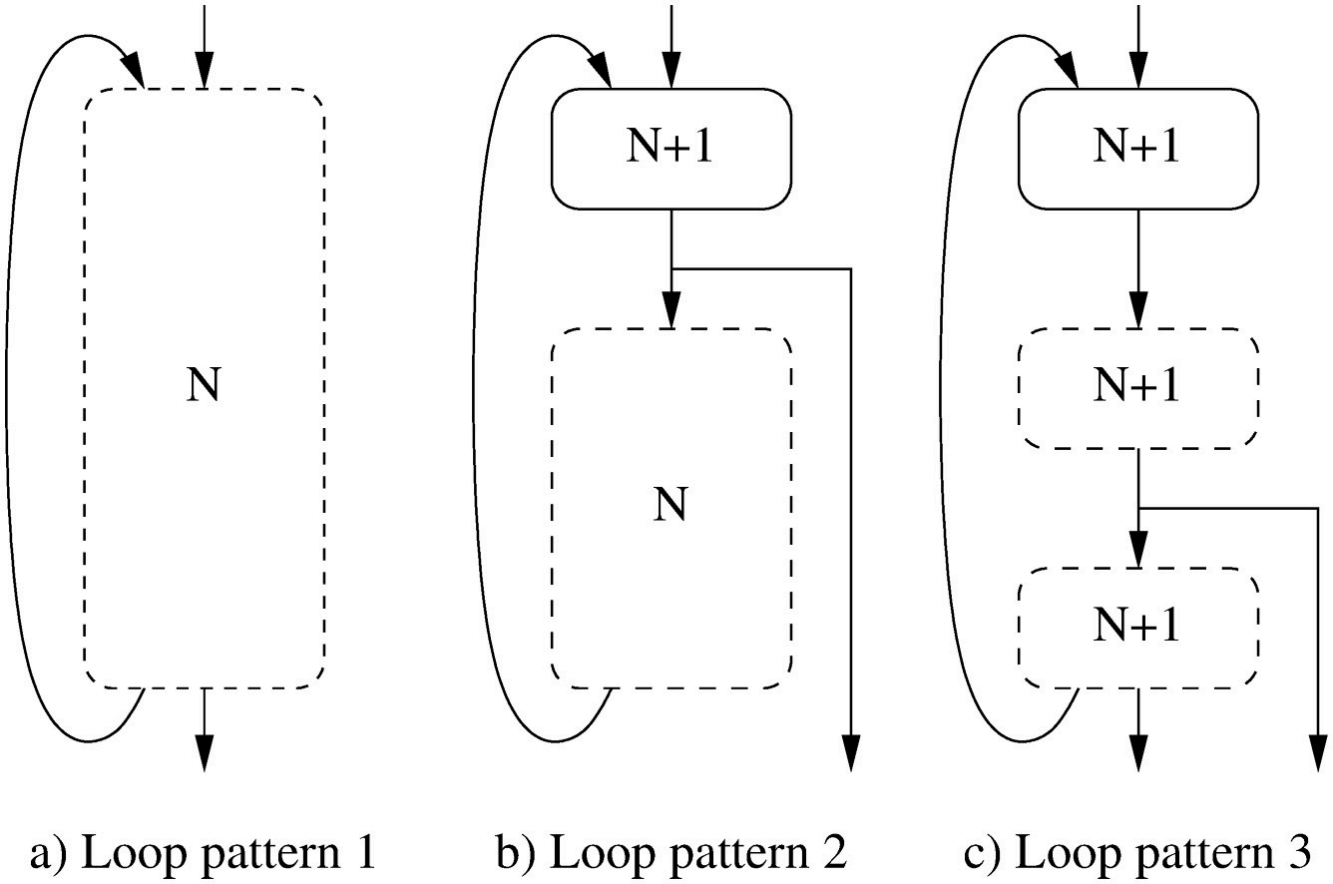
Several loop patterns found in binary code.

Also, let us highlight an additional effect found in *petrinet*. As it can be seen, O2 and O3 present a WCET significantly worse than O1. This is due to the loop transformations performed by these optimizations. Such transformations result in loop patterns with an inherent overestimation in the WCET analysis process. Let us dig into this loop-pattern analysis problem. Depending on the source code and optimization level, the layout of basic blocks containing the loop head and body may be completely different. Fig 8 shows several loop patterns, where *N* is the maximum number of executions of the loop body, tagged by the programmer in the source code of our experiments. In this figure, boxes with dashed borders represent one or more basic blocks, whereas a continuous border represents a single basic block. The A pattern is the basic *do-while* loop. There is only one exit and it is in the same basic block that returns to the beginning of the loop. All basic blocks of this loop are executed *N* times at most. The B pattern (*while* or *for* loop) has one exit only, in the entry basic block. If the loop body is executed *N* times, its entry basic block must be executed *N* + 1 times. In other loop patterns, like C, it can be difficult or impossible to known where is the head or the loop body, which may even be interleaved with loop exit conditions, both distributed along several basic blocks. Therefore, a safe approach implies assuming that all involved basic blocks may be executed *N* + 1 times. This safe approach may actually be an overestimation. This is the case for the O2 and O3 binaries of *petrinet* in Fig 7. In O1, this benchmark contains 151 basic blocks (out of a total of 161 in the benchmark) which are executed 2 times each one in the worst case, whereas in O3 it is assumed that they can be executed up to 3 times. This raises the WCET bound to around 3/2, as can be seen in Fig 7.

## Conclusions

In this work we present DLock-MS, an extension of the WCET analysis method Lock-MS. Our goal is to reduce the WCET analysis time of tasks in presence of a lockable instruction cache. Our extension consist of two main improvements. The first one is an algorithm to transform the CFG to a tree structure, required for using Lock-MS as a structure-based WCET analysis method. Such algorithm generates a tree whose associated ILP model can be easily solved by following a reverse branch-and-bound approach. It performs such transformation in a single pass and processing each alternative path a single time. The resulting tree has much less paths to explore than the original CFG, which reduces the WCET analysis time without sacrificing precision for a locked instruction cache. Our second improvement is a loop-based dynamic locking heuristics, triggered on outer loops, that enables to obtain the optimal cache contents for the WCET of each region, i.e. the configuration that minimizes the WCET of each region. It has a very low complexity, reduces the WCET by exploiting more effectively the temporal reuse, and reduces even more the WCET analysis time by isolating the WCET analysis of each region.

Results show that DLock-MS is around 10 times faster than Otawa, a state of the art tool based on AbsInt and IPET. This fast WCET analysis can be very significant in the design process of a real-time system, and it can be an alternative to parametric WCET analysis. Moreover, our analysis can be stopped before its completion, and in such case it still provides a safe WCET and the configuration for the locked cache to guarantee it. That is, any solution of our model is safe, and completion of the analysis guarantees the optimal (minimum) WCET of each region.

Also, we evaluate the effectiveness of DLock-MS, confirming that it reduces the WCET of the former Lock-MS method, and compare the hit ratio of the locked cache to that of a conventional LRU. Our results show very similar hit ratios in all benchmarks, with the lockable cache offering better hit ratios on many of them. This is done with a very simple hardware.

Finally, we study the impact of the optimization levels on the WCET. Compilation without optimizations (-O0) should be discarded, since it generates binaries with WCETs between 3 and 4 times worse than with optimizations. In general, O3 generates the binaries with the lowest WCETs, but the other optimization levels are also very effective. However, aggressive optimizations in certain benchmarks may result in a significant increment of the WCET. Such optimizations may change the loop patterns so that the WCET analysis method may be forced to assume additional loop iterations.

## Restrictions

It is strictly prohibited to use, to investigate or to develop, in a direct or indirect way, any of the scientific contributions of the authors contained in this work by any army or armed group in the world, for military purposes and for any other use which is against human rights or the environment, unless a written consent of all the authors of this work is obtained, or unless a written consent of all the persons in the world is obtained.

## Supporting information

S1 File (ZIP)
